# Unravelling the distribution of vectors of major vector-borne diseases in Koshi Province of Nepal: A concern of expansion in diverse geo-ecological and climatic regions

**DOI:** 10.1371/journal.pntd.0013188

**Published:** 2026-05-29

**Authors:** Lalita Roy, Surendra Uranw, Raja Ram Pote Shrestha

**Affiliations:** 1 Tropical and Infectious Disease Centre, B. P. Koirala Institute of Health Sciences, Dharan, Nepal; 2 Department of Internal Medicine, B. P. Koirala Institute of Health Sciences, Dharan, Nepal; 3 World Health Organization, Country Office, Kathmandu, Nepal; Beijing Children’s Hospital Capital Medical University, CHINA

## Abstract

**Background:**

Vector-borne diseases (VBDs), including malaria, visceral leishmaniasis, lymphatic filariasis, Japanese encephalitis, and dengue, are major public health concerns and are either slated for elimination or projected for control in Nepal. One of the major challenges in controlling these VBDs is halting their emergence and expansion in diverse geo-ecological and climatic regions. In this study, we collected vectors of major VBDs to assess their distribution, diversity, and associations with ecological variations and to provide an updated understanding of the current situation.

**Methodology/principal findings:**

A descriptive cross-sectional survey was conducted in five districts of Koshi Province, eastern Nepal, during May and June 2023 to collect vectors across three distinct geo-ecological and climatic regions: mountains, hills, and lowlands, situated at altitudes ranging from 98 to 1,274 meters. Adult mosquitoes and sand flies were captured using CDC miniature light traps, BG-Sentinel traps, and manual aspirators. We fitted generalized linear models (GLM) with a negative binomial distribution to assess the association between vector abundance and geo-ecological and climatic variables for two vector species (*Culex quinquefasciatus* and *Phlebotomus argentipes*). We found the malaria vector, *Anopheles annularis*, the lymphatic filariasis vector, *Cx. quinquefasciatus* and the visceral leishmaniasis vector, *Ph. argentipes*, across all three geo-ecological regions. Other vectors of the malaria parasite, *An. pseudowillmori* and *An. willmori*, and Japanese encephalitis vector *Cx. tritaeniorhynchus* were recorded only in hilly districts. Mean temperature and rainfall had a positive effect on *Cx. quinquefasciatus* density, but a deleterious effect on *Ph. argentipes. Culex quinquefasciatus* and *Ph. argentipes* were captured in higher abundance at the household level in the hills (IRR = 1.23 and IRR = 13.00, respectively) and mountains (IRR = 1.96 and IRR = 4.00, respectively) compared with the lowlands.

**Conclusion:**

Two major vectors, *Cx. quinquefasciatus* and *Ph. argentipes* were indiscriminately present in all geo-ecological regions. Climatic variables seemed conducive to vector survival, distribution, and growth across diverse altitudes from the lowlands to the high hills and mountains. Our findings highlight the need for the VBDs control programme to implement regular monitoring, strengthen existing surveillance systems, and support evidence-based planning and implementation of vector control interventions across wider geo-ecological regions to prevent disease transmission.

## Author summary

The Government of Nepal is committed to the elimination and control of major vector-borne diseases: malaria, visceral leishmaniasis, lymphatic filariasis, Japanese encephalitis, and dengue as public health problems by 2030. These diseases, once endemic to the lowlands with tropical and subtropical climates, have spread across a wider geographic range, including the hills and mountains. These high-altitude areas were considered unsuitable for vector survival and pathogen transmission. Considering the impact of climate change in creating favorable environments for the establishment of viable vector populations capable of pathogen transmission, thereby threatening efforts toward VBD control and elimination, we conducted a cross-sectional survey to explore vector diversity across varied geo-ecological regions. We collected vectors from five districts located at different altitudes and characterized by diverse geography. Major vectors responsible for the transmission of pathogens causing lymphatic filariasis and visceral leishmaniasis were abundantly present across all geo-ecological regions, whereas vectors of malaria parasites and dengue virus were sparsely distributed across fewer areas. This study also examined the feasibility of an integrated vector survey to maximize the use of available resources for entomological investigations. Notably, this study provides updated information on vector diversity in areas with VBD cases and recommends that authorities enhance routine monitoring, strengthen existing surveillance systems, and adopt evidence-based planning and implementation of vector control interventions to prevent disease transmission across broader geo-ecological regions.

## Background

Nepal is endemic to at least six vector-borne diseases: malaria, visceral leishmaniasis (VL), lymphatic filariasis (LF), Japanese encephalitis (JE), dengue, and scrub typhus [1]. These diseases are prevalent among the poorest and most marginalized populations, who are often deprived of adequate resources for effective management. During the late 20^th^ century, malaria was endemic in geo-ecological regions below 1,200 m asl [2,3], and VL was endemic in regions situated below 600 m asl [4–6]. Lymphatic filariasis, JE, and dengue were also prevalent in the lowlands [7–9]. In recent years, these VBDs have spread to other geo-ecological regions located at higher altitudes: hills and mountains [10–13]. These regions were once considered unsuitable for vector survival, and pathogen transmission was therefore not anticipated [14–16]. According to the Department of Health Services’ Annual Report 2022-2023 (Nepali calendar year 2079/80), published by the Ministry of Health and Population, a total of 533 confirmed malaria cases were recorded, and the majority of which originated from districts situated in the far-western lowland region [17]. A total of 310 VL cases were reported, mostly from hilly districts. Morbidity data showed 41,535 cases of LF spread over 63% of the total 77 districts of Nepal, encompassing ecological zones from the lowlands to highland mountainous regions. Confirmed JE cases were only 76; however, the disease was found to spread across all districts except 10 in Nepal. An overwhelming number of dengue cases (56,338) were reported from all over Nepal in 2022, creating an outbreak-like situation. Another VBD, scrub typhus, was reported across the country, affecting 9,243 people [17].

The geographical shift in the distribution of VBDs is attributed to several factors, among which climate change is considered the most important. Vectors of these major vector-borne diseases are best adapted to tropical and subtropical climates, with temperatures ranging between 20 – 30 ºC [18–24], relative humidity of 70 – 90% [20,25–27] and annual rainfall of 1,500 mm – 2,000 mm [2,16,28,29]. Optimal climatic conditions maintained in various geographical regions may be due to the effect of climate change [15,19,30–33], which are well suited for vector survival and growth, maintaining the transmission cycles, and thereby leading to the wider spread of pathogens, vectors, and vector-borne diseases [15,30,34,35]. The situation ultimately poses a threat of large-scale epidemics in relatively naïve, susceptible populations, leading to overburdened and unprepared health systems for VBDs control and consequently low-quality health care [15,26,36].

Over the past decades, Nepal has experienced noticeable climate change, especially in two crucial climatic variables: temperature and precipitation. There is an observed increase in temperature of 1.5 °C over the last two and a half decades in Nepal, compared with 0.6 °C at the global level [37]. Similarly, precipitation has increased significantly by 5.3% per decade over the past six decades, with a more rapid increase since the mid-1980s [38]. Recent climatic data have demonstrated an increase in rainfall with altitude on the windward side and a decrease on the leeward (downwind) side in the hills and mountains. Analysis of rainfall shows that the average annual rainfall is 1,883.8 mm in the lowlands (below 1,000 m elevation) and 1,959.6 mm in the highlands (above 1,000 m elevation). The review suggests that July receives the highest rainfall (pre-monsoon to monsoon period), and November (post-monsoon) receives the lowest [39].

Although substantial information is available on VBD reports across different geo-ecological and climatic regions of Nepal, comprehensive data on the occurrence and bionomics of vectors are still lacking. Inadequate vector surveillance data remain a major limitation to effective planning and implementation of vector control programmes in Nepal. Integrated vector monitoring and surveillance is one step closer to the planning and execution of integrated vector management (IVM) and also provides economic benefit to vector control programmes for major VBDs [40].

In this survey, we collected baseline information on vectors of major VBDs, particularly malaria, VL, LF, JE, and dengue across different geo-ecological and climatic conditions in selected districts of Koshi Province, situated in eastern Nepal. The abundance of vector and non-vector species, their associations with the geo-ecological factors (mountains, hills, lowlands and housing conditions) and climatic variables (temperature, rainfall and relative humidity), as well as spatial relationship between vector abundance and VBD occurrence in the study areas, were also assessed.

## Methods

### Ethics statement

Ethical approval to conduct this study was obtained from the Ethical Review Board of the Nepal Health Research Council (NHRC), Kathmandu, Nepal (268/2022P) and the ethical review committee of the WHO Southeast Asia Regional Office, New Delhi, India. Oral and written consent was obtained from the head of households to obtain permission to keep light traps in their homes and to use their cattle for cattle-baited traps.

### Survey sites

**Fig 1:**
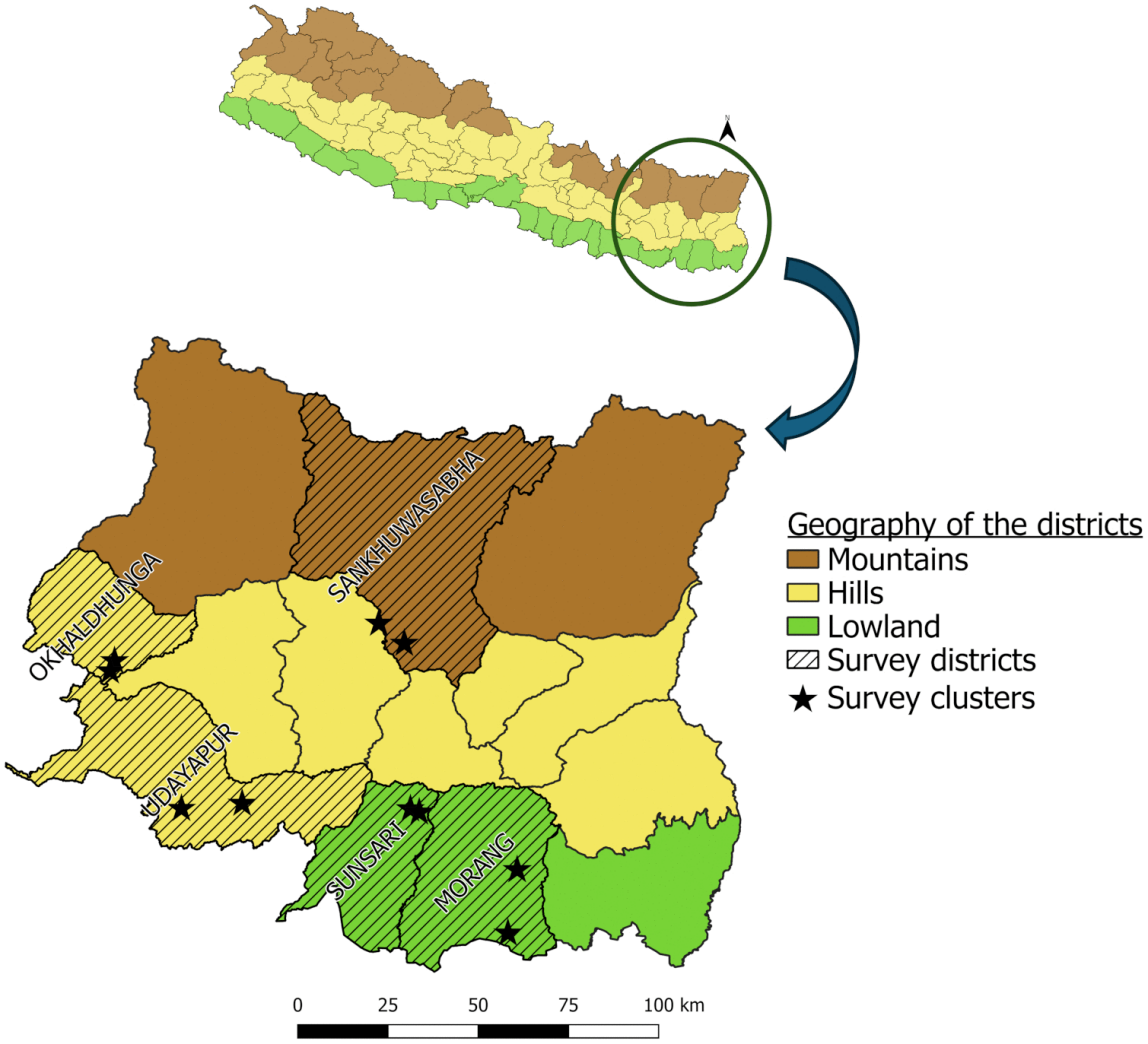
Location of vector survey districts and clusters in Koshi Province, Nepal, 2023. The map was produced with QGIS version 3.36 with an open-access shapefile. (https://opendatanepal.com/datasets/new-political-and-administrative-boundaries-shapefile-of-nepal).

Nepal is topographically divided into three ecological regions from north to south: the high mountains, hills, and lowlands, also known as “terai” (Fig 1). This topography is also reflected by geographical and socio-cultural diversity. The country is administratively divided into 7 provinces and 77 districts. Despite being known for its mountainous terrain, over 80% of the population (approximately 30 million) lives in the lowland regions, which harbor most of the tropical and subtropical diseases. We reviewed the epidemiological surveillance data of the Epidemiology and Disease Control Division (EDCD), Ministry of Health & Population, Government of Nepal, and the VBD patient database of the Koshi Province for the period of 2020 – 2022. All VBD cases from the last three years who were registered in different districts of Koshi Province as their place of residence at the time of admission were listed. The survey districts, namely Sunsari, Morang, Udayapur, Sankhuwasabha, and Okhaldhunga are endemic to visceral leishmaniasis, malaria, and lymphatic filariasis. We retrieved a cumulative total of 101 past VL cases in the document review, including 5 from Sunsari and Sankhuwasabha, 11 from Morang, 12 from Udayapur, and 68 from Okhaldhunga. Only one malaria case was reported from Morang in 2020. Lymphatic filariasis cases were reported from all five districts, but they were not from the survey clusters (villages). Dengue fever was an emerging threat in all these survey districts. These five districts are representative of the three geo-ecological regions: Morang and Sunsari in the lowlands, Udayapur in the low-mid hills, Okhaldhunga in the high hills and Sankhuwasabha in the mountainous region (Fig 1 and Table 1). In each district, we selected two clusters (villages) based on predefined criteria: (i) reports of at least one of the major VBD cases since 2020, (ii) accessibility on foot, and (iii) willingness to support by local health authorities and community. The districts and clusters for vector survey are presented in Table 1.

**Table 1:** Details of the vector survey districts and clusters in Koshi Province, Nepal.

SN	Districts	Ecological region	Municipality	Ward number	Cluster name	Number of total HH*	VBD
1	Morang	Lowlands	Rangeli	6	Godhi tole	70	VL
			Pathari Sanischare	7	Mayalu chowk	55	Malaria
2	Sunsari	Lowlands	Dharan	8	Arun tole	57	Dengue
			Dharan	15	Khoriya basti	78	Dengue
3	Udayapur	Hills	Triyuga	6	Deudi Purano tole	85	VL
			Chaudandigadhi	10	Devdhar	58	VL
4	Okhaldhunga	Hills	Manebhanjyang	5	Fedigaun	40	VL
			Manebhanjyang	6	Sokmatar	48	VL
5	Sankhuwasabha	Mountains	Khandbari	9	Sanguritole	35	VL
			Chainpur	10	Makpa	54	VL

Note: HH* - Households; VBD – vector-borne disease; VL – visceral leishmaniasis.

### Field survey

#### Housing characteristics and geo-ecological information.

In each cluster, 10 households (human dwellings with or without cattle sheds), representing 10 – 20% of the cluster’s total households, were selected using a semi-random approach by choosing every third to fifth household, with preference given to houses with cattle sheds where possible. These households were primarily targeted for mosquito and sand fly collections. Surveyed households were geo-referenced using a Global Positioning System (GPS) device to record the longitude, latitude, and altitude of each house. Each household head was interviewed to collect information on housing structures, the presence of cattle and other domestic animals, surrounding vegetation types, and nearby water bodies.

#### Climate data.

Climatic data such as daily records of rainfall, relative humidity (%), minimum and maximum temperature (°C) for the period of one year (July 2022 – June 2023) for each survey cluster were obtained from the nearest meteorological stations of the Department of Hydrology and Meteorology, Government of Nepal. The meteorological stations were located within 10 km of the survey clusters in the lowlands and within 5 km in the uplands (hills).

#### Collection of vectors.

A descriptive cross-sectional entomology survey was conducted in May and June 2023. The timing of the survey purposively coincided with the beginning of the first annual peak of vector density in the lowlands of Nepal [29,41,42]. Adult and immature stages of vector and non-vector species were collected from 10 selected households within the survey clusters, adjacent vegetation and water bodies, using various methods described below.

i. Adult *Anopheles* and *Culex* mosquitoes (vectors of the malaria parasite, LF, and JE) were collected both from indoor and outdoor human dwellings, cattle sheds, and mixed dwellings (where humans and animals share the same roof in a structure) using mouth aspirators. Two well-trained entomology technicians, having experience in mosquito and sand fly collection for 25 years, collected resting mosquitoes from dwellings by thoroughly searching for all possible places like beams of roof and walls, behind the curtains, corners of the rooms, poles of cattle sheds, and other potential hiding places for 15 minutes in early morning during 0500 hrs to 0700 hrs, in each cluster for two consecutive mornings. Similarly, one cattle-baited trap (CBT), set by covering a single cow with a 4 ft × 8 ft × 4 ft commercial bed net, was used for outdoor mosquito collection outside the household or nearby vegetation area in each cluster. The mosquitoes that entered, landed on, and fed on the cattle were captured by two insect collectors using mouth aspirators for 15 minutes during peak hours of mosquito-biting activity in the evening, which was around 2100 hrs to 2200 hrs [43,44] on day one and day two, and 0500 hrs to 0600 hrs the next consecutive mornings. For safety reasons, CBT was kept outside near the owner’s household. Additionally, Centers for Disease Control and Prevention (CDC) light traps (LT) (Model 512, John W. Hock Company, Gainesville, FL, USA), mainly intended for sand fly collection, were also evaluated for adult mosquito collection.
ii. *Aedes* mosquitoes (vectors of dengue, chikungunya, and zika virus) were collected using Biogents (BG) Sentinel traps (Biogents, Regensburg, Germany) equipped with BG-lure. Five such traps were placed per cluster outdoors, near the households and vegetation, spaced approximately 10 – 20 meters apart to ensure maximum coverage of the cluster [16,45,46]. These traps were also assessed for the outdoor collection of mosquitoes belonging to other genera and sand flies.
iii. Sand flies (vectors of kala-azar): In each cluster, 10 CDC LTs were installed in 10 selected households, one in each. Each light trap was placed either in a human dwelling or in a cattle shed within the household to collect mosquitoes and sand flies. The actual number of LTs in cattle sheds in each cluster varied from zero to five, depending on the availability of such structures, and the remaining were kept in human dwellings. Each light trap was installed one inch away from the wall and 6 inches above the ground in a corner of the main sleeping room or cattle shed and operated from 1800 hrs the evening to 0600 hrs the next morning [29,41,47,48]. The same process was repeated for the next day to complete two consecutive nights of collection. Additionally, resting sand flies were actively searched and captured using mouth aspirators by two well-trained entomology technicians from cracks and crevices on walls, corners of the rooms within human dwellings and cattle sheds, bases of poles and cattle feeding troughs, and around the rat holes. They spent approximately 15 minutes in each household where LTs were installed, conducting aspirator collections for two consecutive mornings [41,47].

Mosquitoes and sand flies were collected and dry-preserved in tubes with silica gel and labeled with cluster code, site, and collection method. The tubes were transported to the entomology laboratory at B.P. Koirala Institute of Health Sciences, Dharan, Nepal. Mosquitoes and sand flies were identified up to species level using regional keys [27,49–56], a stereoscope, and a light microscope. After taxonomic identification, mosquitoes were dry-preserved in tubes with silica gel, while sand flies were preserved in tubes with 80% ethanol. Specimens were stored by species and sex for each cluster.

#### Immature stage survey.

A detailed survey targeting the immature stages of *Aedes* mosquitoes was conducted in two wards of Dharan sub-metropolitan city in Sunsari. First, the number of dengue fever cases registered at B.P. Koirala Institute of Health Sciences, the tertiary health care centre, was analyzed. Subsequently, wards 8 and 15, which reported an increasing number of cases in May 2023, were selected for the survey [57]. Larval and pupal sampling methods were adapted from the standard operating procedure (SOP) and guidelines developed by WHO [58,59]. Randomly selected houses in these wards and public places were systematically searched, both indoors and outdoors, for water-holding containers and the presence of mosquito larvae and pupae. Overhead tanks were not searched due to inconvenience and safety reasons. Besides the water-holding containers for household purposes, discarded plastic and metal containers, tyres, flower vases, plates kept under flowerpots, kitchen gardens, mud pots, gallons, tree holes wherever possible, and any type of utensils that can hold water were also searched for the presence of immature stages. Positive containers were sampled. Larvae and pupae were collected and transported to the entomology laboratory for further rearing to the adult stage, after which they were identified to species level.

### Data management and analysis

All data collected in the field were entered in databases made in Epi Info version 3.5.1 (Centers for Disease Control and Prevention, Atlanta, Georgia, USA) [60]. Descriptive analyses were performed for household characteristics and climatic data (temperature, rainfall, and relative humidity). Abundance and species richness (S) of vector and non-vector species were reported as absolute numbers. Species diversity and dominance or uniformity in distribution of the species at the district level were represented in Shannon-Wiener diversity index (H’) and Pielou’s evenness index (J) [61,62]. H’ and J are calculated using the function ‘diversity’ from a package “vegan” [63] in R version 4.4.2 (R Core Team, Vienna, Austria) [64]. Mathematical calculation was done using the following indices:

(a) Shannon-Wiener diversity index (H’) = -Σp_i_ * ln(p_i_)

Where, Σ = sum, ln = Natural logarithm and p_i_ = n_i_/N (n_i_ = the number of individuals of a species and N = Total number of individuals)

(b) Pielou’s evenness index (J) = H’/ln(S)

Where H’ = Shannon-Wiener diversity index and S is the total number of species in a sample

Interpretation of Shannon-Wiener diversity index (H’) was done as the higher the value of H’, the higher the diversity of species, and the lower the value, the lower the diversity, and if the value of H’ is 0, then only one species is present in that community. Pielou’s evenness index (J) ranges from 0 to 1. The higher the value of J, the higher the level of evenness in the abundance of different species present in a particular community, while a lower value represents either one or only a few species are present in abundance. A landscape map was prepared to illustrate the relative abundances (proportions) of vector species across survey districts and elevations. *Stegomyia* indices (HH index, container index, Breteau index and pupae per person) [58,65–67] were calculated for the immature stages of *Aedes* mosquitoes collected from the urban area of Dharan sub-metropolitan in Sunsari.

As the mosquito and sand fly counts at household level were over-dispersed and had shown non-normal distribution, with variances exceeding the mean values, we fitted generalized linear models (GLM) with a negative binomial distribution to assess the association of the vector abundance in function of the explanatory variables like ecological regions, method of collection, collection sites, climatic variables, household structures, and surrounding ecological features. Spearman’s correlation was assessed between each vector species and the mean temperature (°C), mean relative humidity (%), and cumulative rainfall (mm) of the preceding one month and the month when the survey was conducted (i.e., April and May 2023) before incorporating them into the model. The model was fitted separately for each vector species. The vector species, *An. annularis, An. pseudowillmori, An. willmori, Cx. tritaeniorhynchus, Ae. aegypti* and *Ae. albopictus* were not included in the final model due to their low number of collections across the survey sites, to avoid unstable parameter estimates and violation of model assumptions [68,69], these species were therefore documented descriptively to document their presence and altitudinal distribution. Hence, the model was fitted with only two vector species with plausible collections, *Cx. quinquefasciatus* and *Ph. argentipes*. The calculation was performed using the function ‘glm.nb’ from the R package “MASS” [70]. Results of the analysis are presented as an incidence rate ratios (IRR) and confidence intervals (CI) at 95%.

The incidence rate and vector abundance gradient map for LF and VL were constructed using QGIS version 3.36 (QGIS Development Team, Switzerland) [71]. The disease incidence rates for LF and VL were calculated per 10,000 population at the district level from the available data and the national surveillance data collected in 2022 and 2023. The association between vector abundance and the presence of VBDs at the district level was analyzed with the same GLM method as explained above, and the outcomes were reported as IRR with 95% confidence intervals.

## Results

### Characteristics of districts, households and surroundings in the survey clusters

The elevations of the survey clusters ranged from 98 m asl in one of the lowland clusters in Morang to 1,274 m asl in a cluster situated in a high hill in Okhaldhunga. The key characteristics of the survey districts, the households where LTs were kept and their surrounding areas are shown in Table 2.

**Table 2:** Key characteristics of the survey districts and households in Koshi Province, 2023.

General characteristics	Morang	Sunsari	Udayapur	Okhaldhunga	Sankhuwasabha
Ecological region	Lowland	Lowland	Low-mid hills	High hills	Mountain
Urbanization of clusters	Rural	Urban	Rural	Rural	Rural
Altitude of survey clusters (m asl) (min-max)	98-139	309-323	437-632	1,155-1,274	832-1,011
Endemic for VBDs*	Yes	Yes	Yes	Yes	Yes
**Household characteristics**	**N = 20 (%)**	**N = 20 (%)**	**N = 20 (%)**	**N = 20 (%)**	**N = 20 (%)**
Type of roof					
Cement	3 (15%)	0 (0%)	0 (0%)	0 (0%)	0 (0%)
Thatch (Straw and bamboo)	0 (0%)	2 (10%)	1 (5%)	6 (30%)	9 (45%)
Tiles	0 (0%)	0 (0%)	1 (5%)	0 (0%)	0 (0%)
Tin	17 (85%)	18 (90%)	18 (90%)	14 (70%)	11 (55%)
Type of wall					
Cemented	8 (40%)	6 (30%)	4 (20%)	5 (25%)	1 (5%)
Unplastered brick	1 (5%)	2 (10%)	1 (5%)	2 (10%)	0 (0%)
Mud	11 (55%)	10 (50%)	15 (75%)	13 (65%)	19 (95%)
Tin	0 (0%)	2 (10%)	0 (0%)	0 (0%)	0 (0%)
Type of Floor					
Cement	9 (45%)	10 (50%)	6 (30%)	5 (25%)	3 (15%)
Earthen	11 (55%)	10 (50%)	14 (70%)	15 (75%)	17 (85%)
Presence of ventilation	3 (15%)	7 (35%)	2 (10%)	4 (20%)	1 (5%)
Household with cattle shed	8 (40%)	1 (5%)	12 (60%)	15 (75%)	13 (65%)
Presence of cattle	7 (35%)	0 (0%)	10 (50%)	12 (60%)	11 (55%)
Presence of cow dung	7 (35%)	3 (15%)	12 (60%)	15 (75%)	14 (70%)
Presence of goats	9 ((45%)	5 (25%)	9 (45%)	18 (90%)	14 (70%)
Presence of pigs	7 (35%)	2 (10%)	5 (25%)	4 (20%)	12 (60%)
Presence of agricultural field	18 (90%)	2 (10%)	18 (90%)	10 (50%)	18 (90%)
Presence of vegetable field	14 (70%)	7 (35%)	15 (75%)	14 (70%)	14 (70%)
Presence of mixed orchard	20 (100%)	13 (65%)	16 (80%)	18 (90%)	19 (95%)
Presence of river nearby	10 (50%)	14 (70%)	18 (90%)	14 (70%)	6 (30%)
Presence of pond nearby	10 (50%)	1 (5%)	9 (45%)	6 (30%)	0 (0%)
**Mean number of domestic animals (± sd) per household**					
Cattle	1.25 (±1.97)	0.05 (±0.22)	2.3 (±3.13)	2.5 (±2.40)	2.35 (±2.41)
Goats	2.05 (±3.3)	1.85 (±4)	1.95 (±3.89)	13.5 (±12.9)	4.2 (±5.32)
Pigs	1.15 (±2.23)	0.15 (±0.49)	0.5 (±1.1)	0.25 (± 0.55)	1.25 (±1.21)

*Visceral leishmaniasis, malaria, lymphatic filariasis, dengue fever.

### Status of climatic variables

#### Temperature.

The average daily maximum temperature of the surveyed clusters across the three different ecological regions varied from 31 °C in the lowlands (Sunsari) to 23 °C in the high hills (Okhaldhunga). The average daily maximum temperature was reported to be approximately 35 °C in June in the lowlands (Morang) compared to 28 °C in the high hills (Okhaldhunga). In the same month, it was observed that there was a temperature variation on an average of 7 °C daily between the lowlands and the highlands or hills. The average daily minimum temperature experienced in the lowlands (Rangeli, a cluster in Morang) was about 9 °C in January, compared with 7 °C in the high hills (Okhaldhunga) in the same month (S1 Fig).

#### Relative humidity.

Average daily relative humidity varied from 55.7% in April to 89.1% in September in the survey clusters. April was marked as the driest month, whereas September was the wettest in terms of moisture present in air. The observed relative humidity varied from 70.2% in the lowlands (Morang) to 80.8% in the high hills (Okhaldhunga). The average annual relative humidity was found to be higher in the high hills (Okhaldhunga) compared to all other surveyed districts (S2 Fig).

#### Rainfall.

Annual rainfall also varied according to ecological regions; the lowest annual rainfall (1,451.6 mm) was observed in a cluster located in the lowlands (Rangeli, Morang) and the highest rainfall (2,037.7 mm) in the mid hills (Udayapur). Higher average daily rainfall was observed between June and September (S3 Fig).

### Entomological findings

#### Abundance and types of vector species.

The total number of mosquitoes and sand flies captured was 3,867, of which vector species comprised 77.4% (n = 2,994). They were morphologically identified and segregated into six genera with 28 species of mosquitoes and two genera with three known species of sand flies. Amongst the captured vector species, a few specimens of the genus *Phlebotomus* (n = 11) could not be identified up to the species level. Variation in the species composition of mosquitoes was evident in surveyed clusters and districts. The diversity index was highest in Okhaldhunga for all species collected (H’ = 1.45) and for the vector species (H’ = 0.57). Species richness for both vector and non-vector species was highest in Udayapur (S = 20). For vector species only, species richness was highest in Okhaldhunga (S = 5). Pielou’s evenness index illustrated that one or a few species were dominant, whereas the remaining species were present with nominal density during the time of collection (Tables 3 and 4).

**Table 3:** Distribution and abundance of vector and non-vector species in 10 clusters across five surveyed districts in Koshi Province, 2023.

Districts	Morang		Sunsari		Udayapur		Okhaldhunga		Sankhuwasabha		Total, n (%)
Clusters	Pathari Sanischare-7*	Rangeli-6*	Dharan-8*	Dharan-15*	Chaudandigadhi-10*	Triyuga-6*	Manebhanjyang-5*	Manebhanjyang-6*	Chainpur-10*	Khandbari-9*	
Ecological region	Lowland	Lowland	Lowland	Lowland	Hills	Hills	Hills	Hills	Mountain	Mountain	
**Species**											
Aedes aegypti**	–	–	5	7	–	–	–	–	–	–	12 (0.31)
Aedes albopictus**	–	–	1	–	–	–	–	–	–	1	2 (0.05)
*Aedes pseudotaeniatus*	–	–	–	–	–	–	1	–	–	–	1 (0.03)
*Aedes sp.*	–	–	–	–	–	–	–	1	–	–	1 (0.03
*Anopheles aconitus*	–	–	–	–	–	1	–	–	–	–	1 (0.03)
Anopheles annularis**	4	1	–	–	–	9	1	–	1	2	18 (0.47)
*Anopheles barbirostris*	–	–	–	–	–	2	–	–	–	–	2 (0.05)
*Anopheles culicifacies*	2	2	2	1	22	62	1	1	1	4	98 (2.53)
Anopheles pseudowillmori**	–	–	–	–	–	–	12	2	–	–	14 (0.36)
*Anopheles stephensi*	–	–	5	1	–	1	–	–	–	–	7 (0.18)
*Anopheles subpictus*	3	66	1	–	–	5	–	–	3	–	78 (2.02)
*Anopheles* UN1	–	–	–	–	–	1	–	–	–		1 (0.03)
*Anopheles vagus*	7	27	–	–	2	29	–	–	–	3	68 (1.76)
Anopheles willmori**	–	–	–	–	–	–	5	–	–	–	5 (0.13)
*Armegeres kesseli*	22	–	–	–	6	18	–	–	–	22	68 (1.76)
*Armegeres kuchingensis*	–	–	–	–	–	1	–	–	–	–	1 (0.03)
*Armegeres subalbatus*	–	2	3	3	1	–	–	–	–	4	13 (0.34)
*Culex bitaeniorhynchus*	1	–	–	–	–	3	–	–	–	–	4 (0.10)
*Culex fuscocephala*	1	–	3	1	–	16	1	–	5	27	54 (1.40)
*Culex infula*	1	–	–	–	–	–	–	–	–	–	1 (0.03)
*Culex mimulus*	–	–	–	–	–	1	–	–	–	–	1 (0.03)
Culex quinquefasciatus**	256	435	66	75	3	1003	7	10	96	718	2,669 (69.02)
*Culex sinensis*	1	–	–	–	–	–	–	–	–	–	1 (0.03)
Culex tritaeniorhynchus**	–	–	–	–	–	2	–	–	–	–	2 (0.05)
*Culex vagans*	–	–	–	1	–	–	–	–	–	–	1 (0.03)
*Culex whitei*	–	2	–	–	–	2	–	–	–	–	4 (0.10)
*Mansonia uniformis*	–	–	–	1	–	2	–	–	–	–	3 (0.08)
*Phlebotomu*s (*Adlerius*) spp.	–	–	–	–	–	–	2	1	–	–	3 (0.08)
Phlebotomus argentipes**	2	6	4	5	2	2	43	174	27	7	272 (7.03)
*Phlebotomus major* s.l.	–	–	–	–	–	–	29	20	3	1	53 (1.37)
*Phlebotomus* spp.	–	–	–	–	–	–	7	4	–	–	11 (0.28)
*Sergentomyia* spp.	42	23	14	153	23	40	18	54	1	29	397 (10.27)
*Uranotaenia* sp. complex							1				1 (0.03)
**Grand Total**	**342**	**564**	**104**	**248**	**59**	**1,200**	**128**	**267**	**137**	**818**	**3,867**

Note: *Number represented the ward number of the municipality; **Vector species.

**Table 4:** Diversity index, evenness index, species richness, and abundance of all vector and non-vector species in five districts of Koshi Province, 2023.

Districts	Both vector and non-vector species				Vector species only			
	H’	J	S	A	H’	J	S	A
Morang	0.96	0.36	14	906	0.10	0.09	3	704
Sunsari	1.23	0.49	12	352	0.51	0.37	4	163
Udayapur	0.90	0.30	20	1,259	0.09	0.07	4	1,021
Okhaldhunga	1.45	0.55	14	395	0.57	0.36	5	254
Sankhuwasabha	0.70	0.23	20	955	0.20	0.14	4	852

Note: H’ – Shannon’s diversity index, J – Pielou’s species richness index, S – Species richness, A – Abundance.

#### Distribution of vector species among the districts and the altitudinal gradient.

**Fig 2:**
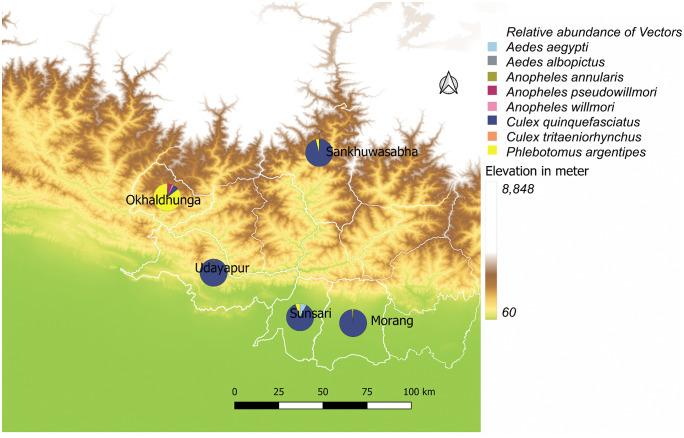
Location where relative abundance (proportion) of the vector species captured in survey districts (Map showing the elevation of the landscape; brown colour- high elevation and green colour- low elevation). The map was produced with QGIS version 3.36 with an open-access shapefile. (https://opendatanepal.com/datasets/new-political-and-administrative-boundaries-shapefile-of-nepal).

**Fig 3:**
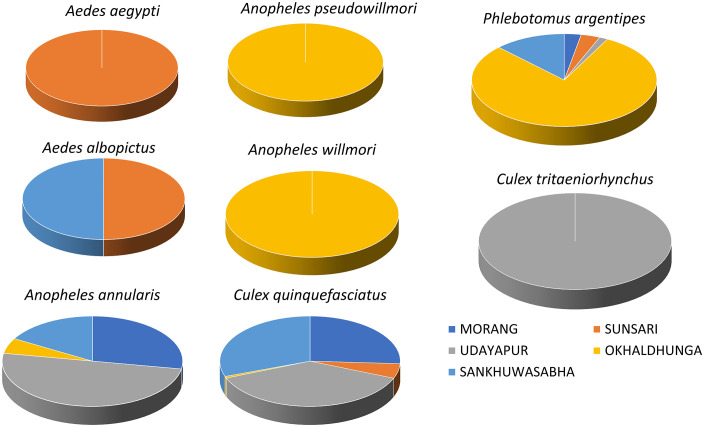
Relative distribution (proportion) of vector species in five surveyed districts, 2023.

One of the known malaria vectors in Nepal, *An. annularis* was captured from all surveyed districts except Sunsari. The other vectors for the malaria parasite, *An. pseudowillmori* and *An. willmori* were also recorded at an altitude of 1,200 m in Okhaldhunga. *Culex quinquefasciatus*, the vector of *Wuchereria bancrofti* microfilariae causing LF in humans, was found at 98 – 1,274 m asl during this survey. Similarly, the vector transmitting the virus causing JE, *Cx. tritaeniorhynchus* was found at 632 m asl in Udayapur. Both vectors, *Ae. aegypti* and *Ae. albopictus* transmitting dengue virus were captured at 318 m asl in Sunsari and only *Ae*. *albopictus* at 832 m asl in Sankhuwasabha. *Phlebotomus argentipes*, the vector of *Leishmania donovani* parasites, was recorded at elevations ranging from 98 to 1,274 m asl. Vector sand fly abundance was four times higher in the surveyed clusters of Okhaldhunga at altitudes above 1,000 m asl. Other suspected sand fly vectors of *Leishmania* spp., *Ph*. *major* sensu lato and *Ph. (Adlerius)* sp. were also recorded in Okhaldhunga and Sankhuwasabha at altitudes ranging from 832 to 1,011 m asl. The details of the vector distribution in the survey districts are illustrated in landscape maps (Figs 2 and 3).

#### Immature stages of *Aedes* mosquitoes.

In two selected wards of Dharan sub-metropolitan city, a total of 434 wet containers in 135 households (including a few public places) with 525 inhabitants were inspected for the *Aedes* larvae and pupae. Of these, 144 wet containers from 81 households were positive for immature stages (Table 5). The household index (HI) was 60% (81/135*100), the container index (CI) was 33.18% (144/434*100), and the Breteau index (BI) was 106.67 (144/135*100). The pupae per person was 0.84 based on 443 pupae collected from the positive containers. These high *Stegomyia* indices (HI, CI, BI, and PPP) were indicative of an outbreak-like situation of dengue fever in the surveyed areas.

**Table 5:** Types of wet containers searched and their contribution to larval productivity.

Types of wet containers	Searched containers (%)	Positive containers (%)	No. of containers with larval density			No. of containers with pupae (pupal count)
			< 10	10 – 50	> 50	
Bowl	6 (1.38)	6 (4.17)	2	3	1	3 (12)
Bucket	66 (15.21)	11 (7.64)	7	3	1	3 (28)
Cement tank	2 (0.46)	2 (1.39)	1	0	1	2 (19)
Ceramic earthen jar	5 (1.15)	5 (3.47)	1	1	3	4 (15)
Coconut shell	2 (0.46)	0 (0.00)	0	0	0	0 (00)
Discarded bottles	15 (3.46)	10 (6.94)	4	4	1	2 (09)
Discarded plastic container	15 (3.46)	2 (1.39)	0	1	0	1 (07)
Discarded Tins	6 (1.38)	0 (0.00)	0	0	0	0 (00)
Ditch	2 (0.46)	1 (0.69)	1	0	0	1 (10)
Drum	242 (55.76)	63 (43.75)	19	16	26	26 (260)
Flower vase	48 (11.06)	27 (18.75)	21	5	1	17 (60)
Gallon	2 (0.46)	1 (0.69)	0	0	0	0 (00)
Mud pot	2 (0.46)	1 (0.69)	0	0	1	0 (00)
Plastic jar	7 (1.61)	5 (3.47)	2	2	1	3 (03)
Tyre	14 (3.23)	10 (6.94)	6	2	1	6 (20)

#### Association of vector abundance with geo-ecological and climatic variables.

Two vector species; *Cx. quinqfasciatus* (n = 2,669, 89.14% and *Ph. argentipes* (n = 272, 9.01%) were considered for the regression analysis as only they were present in plausible numbers for a valid interpretation as compared to the remaining vector species (n = 53, 1.77%). Associations of the geo-ecological and climatic variables are presented separately.

**For *Cx. quinquefasciatus:*** Topography significantly affected the mean abundance of *Cx. quinquefasciatus*. Higher household-level collections were recorded in the hills (IRR = 1.23, CI at 95% = 0.53 – 2.87) and mountains (IRR = 1.96, CI at 95% = 0.73 – 5.91) compared with the lowlands. The result also indicated the existence of a higher density of these vectors at higher altitudes. The CDC light trap was found to be an effective method of vector collection compared to the aspirator (IRR = 0.05, CI at 95% = 0.03 – 0.08).

Considering the household structures, high vector density was recorded in the houses with tiled roofs, mud walls and cemented floor than other types of roofs, walls, or floors (Table 6). Vector density at household-level was lower in well-ventilated rooms (IRR = 0.36, CI at 95% = 0.14 – 1.13) as compared to houses without proper ventilation. We observed an increasing effect on *Cx. quinquefasciatus* density per household in the presence of goats, pigs, agricultural fields, mixed orchards containing a variety of tropical and subtropical plants present near the household, and nearby rivers, ponds and drains (Table 6).

**Table 6:** Association of geo-ecological and climatic factors with *Cx. quinquefasciatus* and *Ph. argentipes* density.

Explanatory variables		IRR (CI at 95%) for *Culex quinquefasciatus*	IRR (CI at 95%) for *Phlebotomus argentipes*
**General variables**			
Ecological region (ref: Lowland)	Intercept	20.80 (12.11 – 40.07)	0.43 (0.20 – 0.92)
	Hills	1.23 (0.53 – 2.87)	13.00 (5.06 – 34.04)
	Mountains	1.96 (0.73 – 5.91)	4.00 (1.31 – 13.38)
Method of collection (ref: Light trap)	Intercept	12.55 (9.07 – 18.04)	1.06 (0.67 – 1.79)
	Aspirator	0.05 (0.03 – 0.08)	0.18 (0.09 – 0.34)
	BG sentinel trap	0.06 (0.01 – 0.60)	0.63 (0.09 – 19.66)
**Household characteristics**			
Type of roof (ref: Tin)	Intercept	28.42 (19.20 – 44.57)	2.08 (1.26 – 3.41)
	Cemented	0.13 (0.02 – 3.41)	0
	Thatched	0.61 (0.25 – 1.81)	2.94 (0.96 – 9.00)
	Tiles	4.57 (0.42 – 11017.67)	0
Type of floor (ref: Earthen)	Intercept	25.61 (16.68 – 42.16)	2.84 (1.68 – 5.25)
	Cement	1.13 (0.52 – 2.64)	0.88 (0.34 – 2.50)
Type of wall (ref: Mud plastered)	Intercept	31.63 (20.84 – 51.28)	2.44 (1.45 – 4.48)
	Cemented	0.62 (0.27 – 1.61)	1.57 (0.57 – 5.17)
	Tin	0.24 (0.03 – 19.27)	0.41 (0.02 – 164.73)
	Un-plastered	0.17 (0.04 – 1.31)	0.82 (0.16 – 11.47)
Ventilation present (ref: No ventilation)	Intercept	30.14 (20.59 – 46.59)	2.99 (1.87 – 5.13)
	Yes	0.33 (0.13 – 1.00)	0.47 (0.15 – 1.96)
**Ecological variables**			
Cattle present (ref: No)	Intercept	31.23 (19.95 – 52.82)	0.98 (0.57 – 1.80)
	Yes	0.64 (0.30 – 1.41)	5.42 (2.34 – 13.05)
Goat present (ref: No)	Intercept	22.91 (13.69 – 42.52)	0.40 (0.20 – 0.83)
	Yes	1.30 (0.60 – 2.77)	11.55 (4.83 – 27.79)
Pigs present (ref: No)	Intercept	2.67 (1.60 – 4.88)	2.67 (1.60 – 4.88)
	Yes	1.06 (0.40 – 3.15)	1.06 (0.40 – 3.15)
Cow dung near the house (ref: No)	Intercept	33.88 (20.74 – 60.79)	0.78 (0.42 – 1.54)
	Yes	0.58 (0.27 – 1.24)	5.92 (2.49 – 14.06)
Agricultural field present (ref: No)	Intercept	12.53 (7.04 – 25.38)	2.56 (1.25 – 6.29)
	Yes	2.71 (1.19 – 5.78)	1.10 (0.39 – 2.83)
Vegetable field present (ref: No)	Intercept	31.25 (17.69 – 62.87)	1.42 (0.70 – 3.35)
	Yes	0.77 (0.34 – 1.66)	2.44 (0.89 – 6.21)
Mixed orchard present (ref: No)	Intercept	23.21 (9.75 – 77.60)	3.36 (1.18 – 15.14)
	Yes	1.17 (0.33 – 3.12)	0.78 (0.16 – 2.24)
River present (ref: No)	Intercept	25.03 (14.34 – 49.41)	1.5 (0.75 – 3.46)
	Yes	1.11 (0.49 – 2.37)	2.31 (0.86 – 5.85)
Ponds present (ref: No)	Intercept	16.82 (11.33 – 26.45)	2.69 (1.63 – 4.82)
	Yes	3.26 (1.49 – 7.93)	1.04 (0.39 – 3.32)
Drains present (ref: No)	Intercept	18.02 (11.72 – 29.68)	3.94 (2.44 – 6.85)
	Yes	2.34 (1.10 – 5.23)	0.14 (0.06 – 0.37)
Ditches present (ref: No)	Intercept	26.75 (17.26 – 44.59)	2.55 (1.49 – 4.79)
	Yes	0.99 (0.46 – 2.27)	1.19 (0.47 – 3.28)
**Climatic variables**			
Temperature (0 °C)	Intercept	0.32 (0.01 – 19.11)	33153(3638.95 – 359832.80)
	Mean temperature (April and May)	1.19 (1.01 – 1.40)	0.66 (0.59 – 0.72)
Relative humidity (%)	Intercept	595.78 (8.30 – 52598.68)	0
	Mean relative humidity (April and May)	0.95 (0.88 – 1.02)	1.29 (1.20 – 1.41)
Rainfall (mm)	Intercept	12.36 (2.36 – 77.42)	3.86 (0.03 – 472.73)
	Cumulative Rainfall (April and May)	1.00 (0.99 – 1.01)	1.00 (0.97 – 1.03)

**For *Ph. argentipes:*** A significant effect of topography has been observed with *Ph. argentipes* density, which was collected almost 13 times higher in hilly and four times in mountainous districts as compared to the lowlands. CDC light traps were found to be a more efficient method of sand fly collection compared to the aspirators (IRR = 0.18, CI at 95% = 0.09 – 0.34). Houses with thatched roofs, cemented walls, earthen floors, and poor or no ventilation showed an increasing effect on the vector density. Other ecological factors, including the presence of cattle, goats, agricultural fields, vegetable fields, rivers, ponds, and ditches, showed an increasing effect on *Ph. argentipes* density at the household level (Table 6).

**Fig 4:**
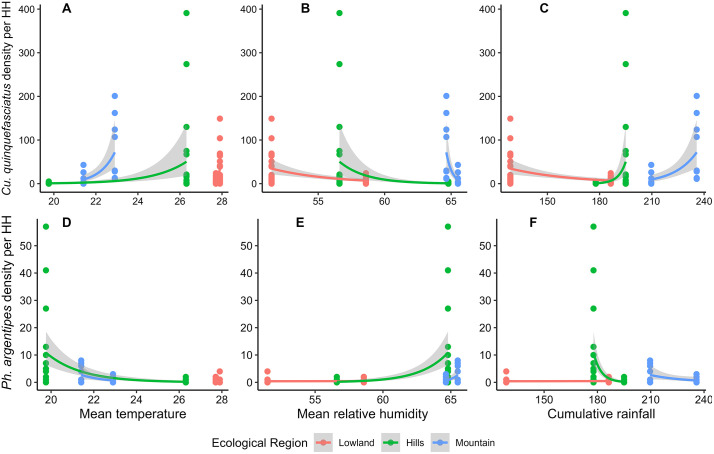
Scattered plots and regression lines showing the effects of climatic variables on vector density in three ecological regions. Panels A, B, and C show the effects of climatic variables on *Cx. quinquefasciatus* density and panels D, E, and F show effects on *Ph. argentipes* density at the household level. Dots represent the data points, line represents the generalized regression line with a negative binomial distribution, and the shaded area indicates the standard error of the regression line.

We also observed a weak but statistically significant positive correlation between *Cx. quinquefasciatus* density and mean temperature (r = 0.38, p < 0.001), and a significant negative correlation with mean relative humidity recorded in April and May (r = -0.32, p < 0.001). A very weak and non-significant correlation was observed with rainfall (r = 0.11, p = 0.27). When these climatic factors were fitted into the model, mean temperature recorded in April and May showed a positive association with vector density (IRR = 1.19, CI at 95% = 1.01 – 1.40). Overall, rainfall had a negligible effect on the vector density; however, when analysed by ecological region, the model showed an increasing effect in the hills and mountains and a decreasing effect in the lowlands. Another climatic variable, relative humidity, had a decreasing effect on household-level vector density across all geo-ecological regions (Fig 4).

*Phlebotomus argentipes* density showed a negative correlation with temperature (r = -0.51, p < 0.001), a weak but significant positive correlation with relative humidity (r = 0.50, p < 0.001), and a negative and non-significant correlation with rainfall (r = -0.10, p = 0.32). While fitted in the model with climatic data of April and May, mean relative humidity showed an increasing effect (IRR = 1.29, CI at 95% = 1.20 – 1.41), mean temperature had a decreasing effect, and cumulative rainfall showed no effect on vector density (Table 6 and Fig 4).

#### Spatial relationship between vector abundance and vector-borne diseases.

**Fig 5:**
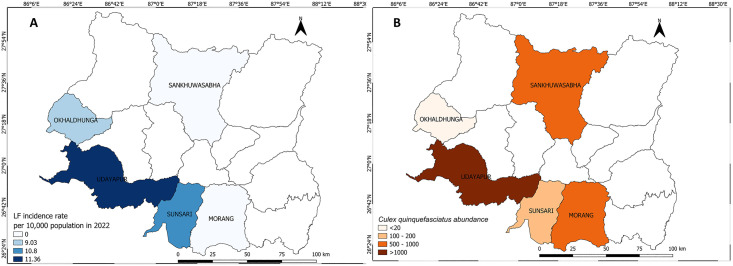
Lymphatic filariasis incidence rate in 2022 (A) and *Cx. quinquefasciatus* abundance (B) during the survey in five study districts in Koshi Province, 2023. The map was produced with QGIS version 3.36 with an open-access shapefile. (https://opendatanepal.com/datasets/new-political-and-administrative-boundaries-shapefile-of-nepal).

**Fig 6:**
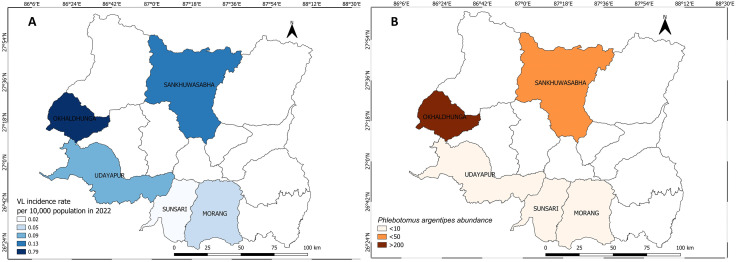
Visceral leishmaniasis incidence rate in 2022 (A) and *Ph. argentipes* abundance (B) during the survey in five study districts in Koshi Province. The map was produced with QGIS version 3.36 with an open-access shapefile. (https://opendatanepal.com/datasets/new-political-and-administrative-boundaries-shapefile-of-nepal).

The high disease incidence for LF coincided closely with the high *Cx. quinquefasciatus* abundance in Udayapur (Fig 5). The LF incidence rate was not available in the national line list for Morang and Sankhuwasabha. A similar pattern was observed for VL; the district with the highest incidence rate also having the highest number of *Ph. argentipes* (Fig 6).

When fitted in the model, the disease incidence for LF was not associated with vector density, possibly due to the absence of data from two districts. However, higher *Ph. argentipes* density was associated with an increased VL incidence rate (IRR = 1.04, CI at 95% = 1.01 – 1.06) at the district level (Table 7).

**Table 7:** Association of vector density with the incidence rates of major VBDs at the district level in Koshi Province.

Explanatory variables		IRR (CI at 95%) for Lymphatic filariasis incidence rate	IRR (CI at 95%) for Visceral leishmaniasis incidence rate
*Culex quinquefasciatus*	Intercept	0.65 (6.01 – 7.13)	–
	Density at the household level	1.00 (1.00 – 1.00)	–
*Phlebotomus argentipes*	Intercept	–	0.18 (0.11 – 0.28)
	Density at the household level	–	1.04 (1.01 – 1.06)

## Discussion

This study represents the first integrated vector survey to generate primary surveillance data on multiple human-biting vector species using vector-specific collection methods across diverse ecological regions. Our study shows the presence and abundance of the major vectors of pathogens causing malaria, VL, LF and dengue across lowland to highland districts situated in different geo-ecological and climatic regions in Koshi Province of Nepal. Diverse mosquito species were recorded in all survey districts, accounting for approximately 15.5% of the total mosquito species reported from Nepal [52,54,72]. During the investigation, adult *Ae. aegypti* and *Ae. albopictus*, the primary vectors of dengue virus, were collected in very low numbers. The *Stegomyia* indices based on the immature stages of *Aedes* spp. in urban habitats of the Dharan sub-metropolitan city, situated at an altitude of 300 m, were high enough to indicate intense dengue virus transmission. Our survey documented well-established populations of *Cx. quinquefasciatus* and *Ph. argentipes* in the hills and mountains, likely facilitated by the socio-ecological conditions and microhabitat of these locations. In this study, we found a positive association of *Cx. quinquefasciatus* density with mean temperature, while relative humidity showed an increasing effect on *Ph. argentipes* density. The observed abundances of the vector species *Cx. quinquefasciatus* and *Ph. argentipes* were epidemiologically significant, as reflected by case records from 2022, one year before the survey, during which 1,515 LF cases and 24 VL cases were reported from the five surveyed districts [17].

The major malaria vector, *An. fluviatilis*, was not recorded from any of the surveyed clusters of the five districts. Other malaria vectors, *An. maculatus* complex species; *An. willmori* and *An. pseudowillmori*, were recorded from one of the high hill districts (Okhaldhunga) above 1,200 m altitude. There are reports of the presence of malaria vectors from high altitudes at 1,300 m up to 2,000 m in previous studies as well [2,73,74]. The abundance of *Cx. quinquefasciatus* from low to high altitudes demonstrated its resilience and adaptability, enabling its survival and establishment across a wide range of geo-ecological and climatic conditions in Nepal [2,16,32,75,76]. Entomological surveillance of the JE virus vector *Cx. tritaeniorhynchus* has been infrequent; however, predictive model analyses have shown that the species is predominantly Asiatic, with highly suitable environments located across Nepal, India, and China, and thus potentially facilitating the spread of the disease throughout the region [77]. In Nepal, JE is present in 63 out of 77 districts, mostly in the lowlands, with occasional outbreaks reported in the mid-hills and mountainous regions [78]. Evidence of local transmission of JE was supported by suitable ecological conditions and the abundance of vector species [2,75,76,79]. Previous studies conducted in Nepal have well documented the presence and abundance of *Ae. aegypti* and *Ae. albopictus* from the lowlands to high hill regions above 2,300 m asl [15,16,26,33,72]. Another significant VBD, VL, is widely distributed in 72 endemic and endemicity-doubtful districts covering all geo-ecological and climatic regions [80], and most of these districts harbor viable vector populations, including those in the high hills and mountainous areas [14,41,81–84]. The results of the present study also indicated well-established populations of *Ph. argentipes* with high densities at high altitudes. Additionally, the presence of other competent sand fly vectors has amplified the threat of VL transmission in high-altitude areas [81]. A similar context of VL transmission, together with evidence of potential vector species in high-altitude regions of bordering states in India [85,86], supports the expansion of the disease into wider geo-ecological zones.

Overall, socio-ecological factors in the surveyed areas played a crucial role in the survival and proliferation of vectors. However, very limited research is available on the assessment of socio-ecological factors influencing vector abundance. A study conducted in an African country demonstrated the association between the presence of windows and fewer mosquitoes indoors [87]. Evidence from studies conducted in India and Brazil has shown that tiled and concrete houses with a high LF case burden were often associated with nearby breeding habitats and surrounding vegetation favorable for the proliferation of *Cx. quinquefasciatus* populations [88,89]. Findings from India and Nepal demonstrated that ecological factors like mud-walled houses, the presence of cattle, goats, pigs, cow dung near the house, nearby vegetation types, and nearby water resources had a direct influence on the abundance of *Ph. argentipes* [90] and thus act as risk factors for VL as well [91]. In line with findings from other parts of Nepal, the most suitable habitats for larvae of *Aedes* species were earthen pots and discarded tyres [33]. Dengue outbreaks were generally associated with high *Stegomyia* indices [92], which was also observed in our investigation.

The current epidemiological and entomological data in Nepal indicate the widespread distribution of major VBDs and their respective vectors. Climate change over the past 40 years has been evident in Nepal through an estimated 0.056 °C rise in the average annual maximum temperature, with increasing warming at higher altitudes [93]. Changes in temperature and rainfall strongly influence the geographical distribution of vectors and associated VBDs [94]. An ecological time-series analysis in Nepal showed a 10.14% rise in VBD-related hospitalizations per 1 °C rise in temperature [95]. Another study projected a 27% and 25% increase in malaria incidence with a 1 °C rise in minimum and mean temperatures, respectively [42]. A 2018 report from the Lancet Countdown on health and climate change showed that the global vectorial capacity for dengue virus transmission by *Ae. aegypti* and *Ae. albopictus* increased by 9.1% and 11.1%, respectively, in 2016 compared with the 1950s baseline [96]. In the context of climate change, an ecological niche model in Nepal predicted the spread of dengue virus transmission to higher altitudes, along with an increase in caseloads [97], which is further supported by this study.

Despite generating valuable baseline information on the distribution and diversity of vectors, the present study has several important limitations. First, the entomological survey was a cross-sectional integrated vector survey conducted in limited areas. Therefore, the findings are not sufficient to generalize the diversity, distribution, and bionomics of vectors across all seasons and provinces in Nepal. Such generalization can only be possible through a study designed to conduct year-round surveillance of vectors across a wider geographical region. A single-time survey could be the reason why malaria vector *An. fluviatilis* was not collected from any of the clusters. Second, the timing of entomological survey was selected based on peak vector-borne disease transmission seasons in the lowlands, as no relevant data were available from the hills and mountains. As vector diversity, distribution, and bionomics in the hills and mountains are also influenced by climatic and ecological factors, determining the optimal timing for vector collection in these regions remains challenging. Prior knowledge of vector seasonality in hilly and mountainous districts is therefore crucial for designing effective surveys. Third, the *Aedes* larval survey was limited to a single district due to logistical constraints, preventing comparative analyses and limiting the generalizability of the results.

In summary, the current study underscores the need for expanded longitudinal, year-round vector surveillance using statistically powered sampling designs across diverse geo-ecological regions to comprehensively characterize the seasonal and interannual dynamics of vector populations, distribution, and diversity. Sustainable integrated vector surveillance at selected sentinel sites across diverse geo-ecological regions is necessary for evidence-based decision-making and the implementation of effective vector control strategies. Habitat reduction and community awareness activities can be conducted alongside dengue vector surveillance. Entomological surveys should also be conducted routinely during the non-transmission season. Xenomonitoring of pathogens in vector populations can be used to assess the risk of pathogen transmission in human populations living in endemic or non-endemic areas. In case of outbreaks of a particular VBD, targeted vector surveillance should be conducted to generate evidence for prompt actions on vector control and management.

## Conclusions

This study provides baseline information on the diversity of vectors of major VBDs and demonstrates well-established vector populations across all geo-ecological regions with varying climatic conditions in Koshi Province, Nepal. The two abundant vector species, *Cx. quinquefasciatus* and *Ph. argentipes*, were indiscriminately present from the lowlands to the highlands (hills and mountains). Overall, the ecological and climatic conditions appear suitable for the survival, distribution, and growth of vector populations. The study also examines the feasibility of integrated vector surveillance rather than disease-specific surveillance as a strategy to enhance the efficient utilization of available resources and funding. In addition, these study findings alert the vector control programmes to implement regular monitoring, strengthen existing surveillance systems, and ensure timely control interventions in diverse areas prone to VBD transmission.

## Supporting information

S1 Fig Average daily maximum, minimum and mean temperatures (ºC) in the surveyed clusters and districts from July 2022 to June 2023. (JPG)

S2 Fig Average daily relative humidity (%) in the surveyed clusters and districts from July 2022 to June 2023. (JPG)

S3 Fig Average daily rainfall (mm) in the surveyed clusters and districts from July 2022 to June 2023. (JPG)

S1 Data A Dataset of vectors and non-vector mosquitoes and sand fly collected from survey districts in detail. (XLS)
