# Supplementary figures and images for: Unravelling the distribution of vectors of major vector-borne diseases in Koshi Province of Nepal: A concern of expansion in diverse geo-ecological and climatic regions
Source: PLoS Negl Trop Dis. 2026 May 29;20(5):e0013188. doi: 10.1371/journal.pntd.0013188 (PMC13235926; doi:10.1371/journal.pntd.0013188)

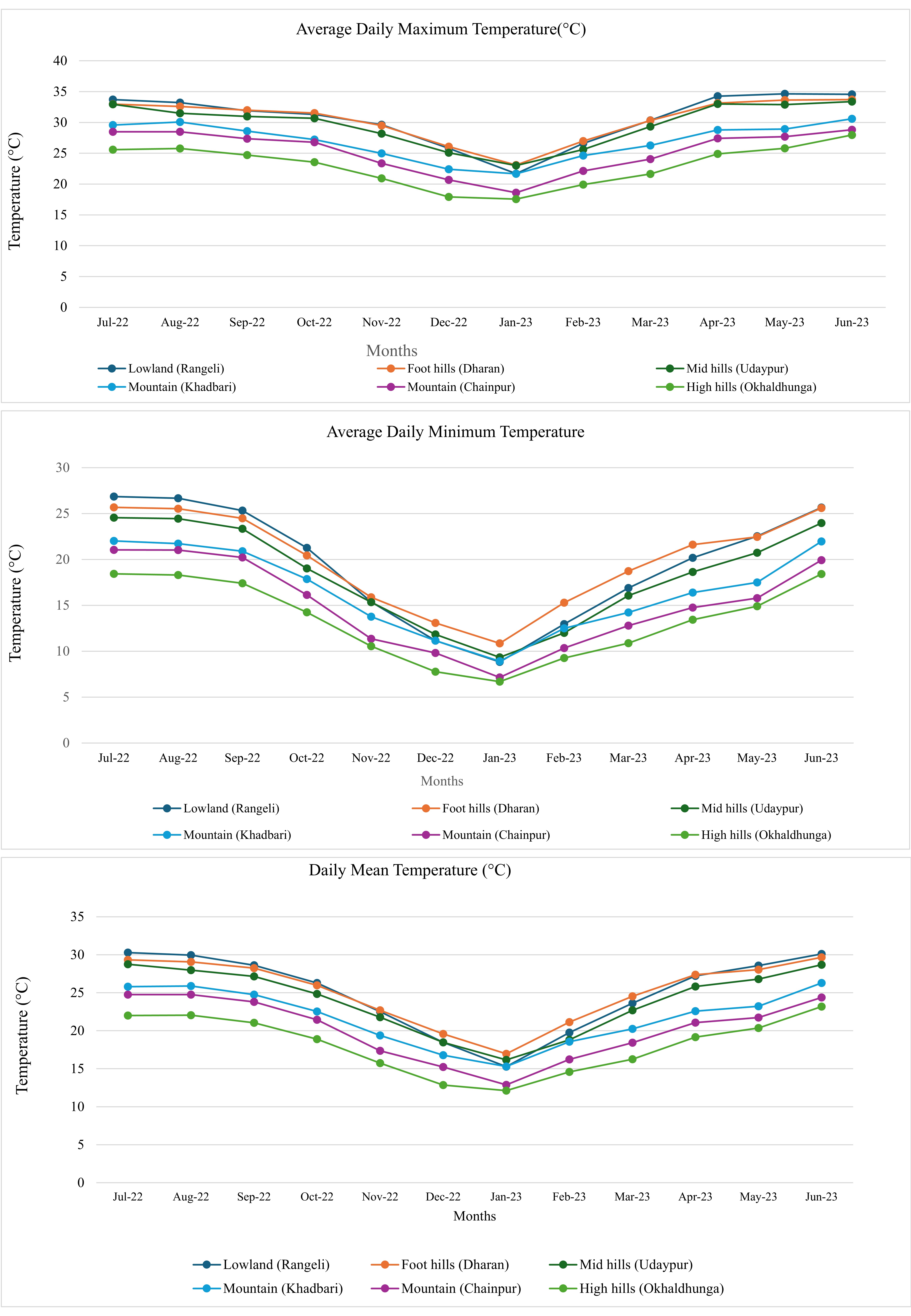

Supplement: S1 Fig — (JPG) [file pntd.0013188.s001.jpg]

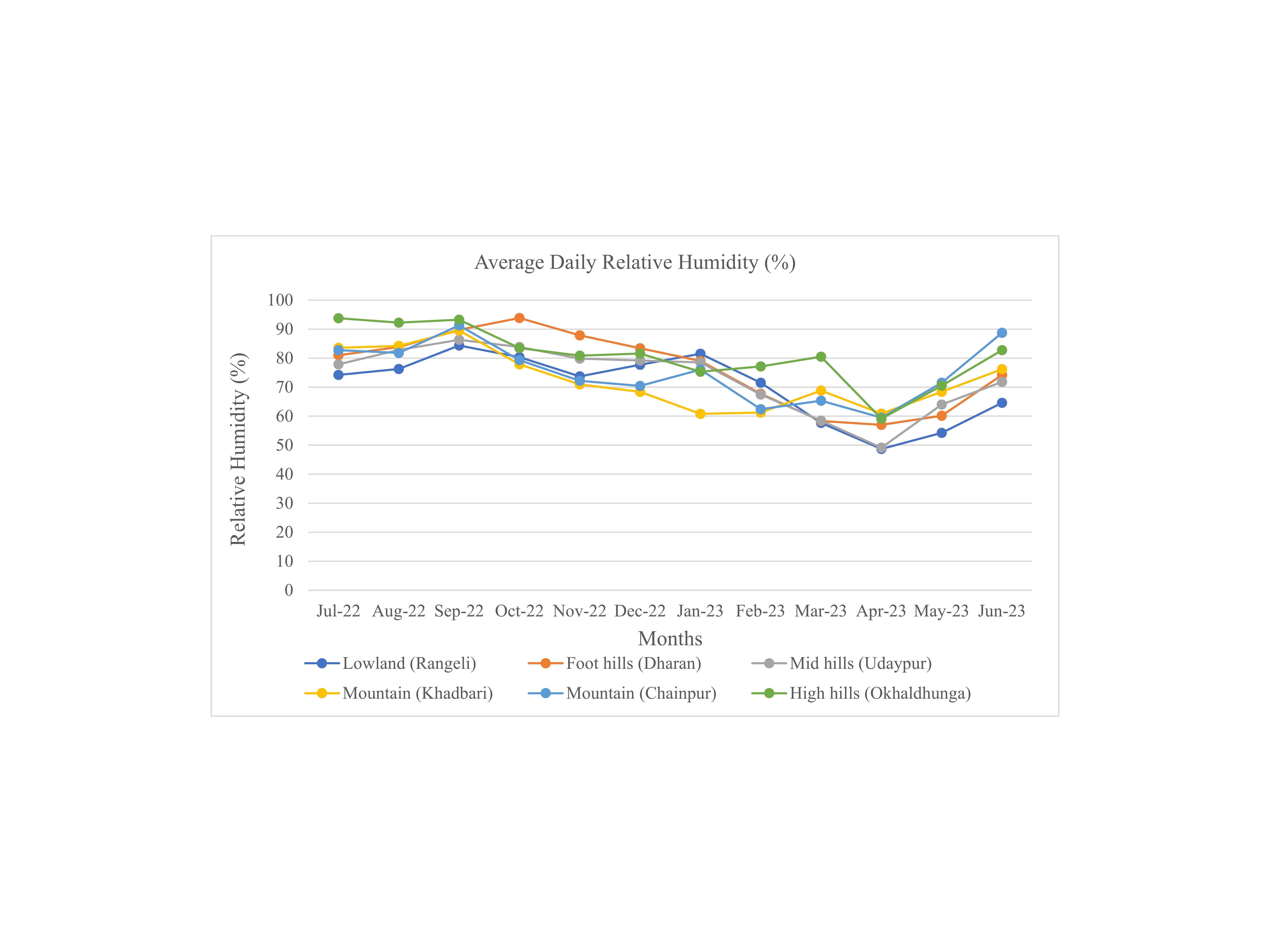

Supplement: S2 Fig — (JPG) [file pntd.0013188.s002.jpg]

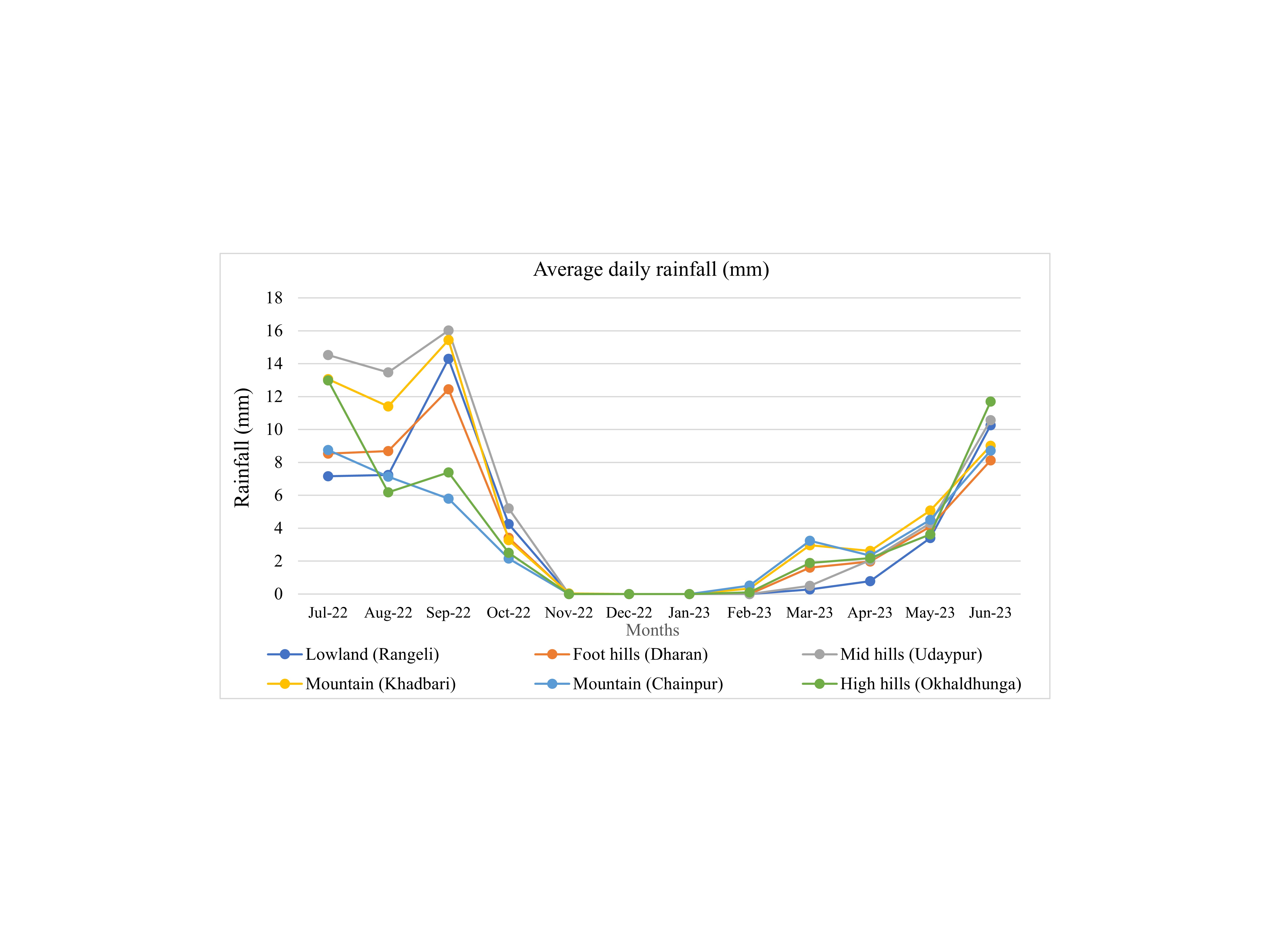

Supplement: S3 Fig — (JPG) [file pntd.0013188.s003.jpg]
